# Development and Validation of the Perceived Authenticity Scale for Cheese Specialties with Protected Designation of Origin

**DOI:** 10.3390/foods10020248

**Published:** 2021-01-26

**Authors:** Katia Laura Sidali, Roberta Capitello, Akhsa Joanne Taridaasi Manurung

**Affiliations:** 1 Department of Business Administration, University of Verona, via Cantarane, 24 1, 37129 Verona, Italy; roberta.capitello@univr.it; 2 Marketing of Food and Agricultural Products Faculty of Agricultural Economics, G-A University of Goettingen, Platz der Goettinger Sieben 5, 37073 Goettingen, Germany; akhsamanurung@yahoo.de

**Keywords:** authenticity scale, genuine, cheese specialty, country-of-origin labels, product identification, stated willingness to consume

## Abstract

Authenticity has become increasingly important in the modern market as consumers seek products more resonant of tradition and originality. This study aimed to develop and validate a perceived authenticity scale for food specialties. Furthermore, this work exposed the causal relationship between authenticity and consumer behaviour, by quantitatively analysing the effects of perception of authenticity and identification with a product on consumers’ willingness to consume the cheese Algovian Emmentaler, an iconic dairy product produced in southern Germany and protected with the designation of origin. Three surveys were conducted over two different timeframes. One served as a pre-test in Germany with a representative sample for the second two in Germany and Italy with a gourmet sample. Both objective authenticity and subjective authenticity were considered, with the former comprising concepts such as whether the respondent was sure of the cheese’s origin and the latter what the cheese embodied. Identification with Algovian Emmentaler was also surveyed. Exploratory factor analysis and confirmatory factor analysis were conducted on the survey data in order to construct an authenticity scale. Based on this scale, structural equation models were constructed. Objective authenticity was found to positively contribute to stated willingness to consume, as well as mediate subjective authenticity, which itself mediated the effects of identification. Subjective authenticity was a large contributing factor to willingness to consume among German consumers, whereas the effects of objective authenticity were higher in Italy compared with the former. Expectedly, identification with Algovian Emmentaler also had a high direct effect on willingness to consume in Germany.

## 1. Introduction

The increasing appeal of traditional, less sophisticated products among consumers on the marketplace has led to an increase in the significance of authenticity [1]. Many studies focusing explicitly on authenticity have been conducted across various domains, including tourism [2,3,4] wines, brands [5,6,7,8,9], business organization [10], advertising [5,6,7,8,9,10,11] and culture [12,13]. In the agrifood sector, worldwide interest in food-related issues is growing, making determining the origin of food of paramount importance [14,15]. According to Kuznesof et al. [16], the perceived authenticity of a product attributes by consumers applies above all to regional food. Moreover, Verbeke et al. [17] state that the perception of better product quality is one of the reasons for the consumption of specialty food that is often branded via quality labels or geographical indications (GIs) such as protected designation of origin (PDO) and protected geographical indication (PGI).

Furthermore, food safety issues, such as bovine spongiform encephalopathy (BSE), foot-and-mouth disease and malpractice among food producers have intensified public sensitivity regarding the origin of food. This has led to a surge in demand for high-quality products and desire for cultural identification [18]. There is, however, a gap in knowledge in various areas of research. Despite the number of studies on authenticity, very few focus on food marketing. This is particularly true for quantitative studies. To the best of our knowledge only a single study, conducted by Sidali and Hemmerling [19], has attempted to measure consumers’ perceived authenticity of a specialty food, which was found to have a positive influence on stated consumption. No study to date has used a similar approach in a cross-country context. Therefore, this study attempts to fill this gap by quantitatively measuring customers’ perceived authenticity of a German traditional specialty cheese in relation to their consumption of it. To achieve this, a perceived authenticity scale was developed using data from online surveys of gourmet consumers that were conducted during two different timeframes and in two different countries, namely Germany, where this cheese originates, and Italy, where lower knowledge of the product is compensated by a higher general culinary awareness. After the validity of the authenticity scale was verified, its effect on German and Italian respondents’ stated willingness to consume the cheese was analysed.

Product authenticity is a keystone of modern marketing [19]. Spiggle et al. [7] observed that an authentic product comprehends the meaning and essence of the tradition it embodies. Authenticity can be treated as a unidimensional, bi-dimensional, or multidimensional construct, with consumers’ desiring uniqueness [5,20,21], naturalness [15,20], country-specific origin [22] or credibility [5,23]. Liao and Ma [22] found that respondents with a high need for authenticity tend to associate it with six characteristics: (1) originality, (2) quality commitment and credibility, (3) heritage and style persistence, (4) scarceness, (5) sacredness and (6) purity. In her study on food authenticity and tourism, Sims [24] claims that consumers’ demand for traditional and local food can also be viewed as linked to a quest for authenticity. Especially on holiday, the consumption of local food and drink products is a way of restoring a more meaningful sense of connection between the consumers and the people and places that produce their food [24]. Sidali and Hemmerling [25] suggest that the best way of surveying authenticity systematically is the multidimensional method. As such, a large number of studies distinguish between subjective authenticity and objective authenticity. The former depends on a consumer’s personal experience with a product, to which a set of values is attached [16,21,23,26,27], as well as the social and environmental issues associated with sustainability, which includes supporting the local economy, fairly compensating local farmers and maintaining biodiversity [28]. Onozaka et al. [29] reported that purchasing behaviours were affected by the perceived availability of local food and perceived effectiveness of their actions, wherein customers believed that they made a difference in public outcomes, such as supporting the local economy. Contrary to subjective authenticity, perceived objective authenticity regards the physical attributes of the product and the production method, whether it is the traditionality of the recipe, the provenance of the ingredients, or the combination of soil and climatic conditions that are peculiar to the production area. Grayson and Martinec [30] recognized physical attributes as indexical authenticity. Moreover, Carcea et al. [15] stated that the perception of food authenticity is important at the emotional level because of the involvement of trust in the buying decision. Tradition and identity also play an important role in how food authenticity is perceived, as part of what makes it authentic is the traditional methods used. Research on perceived authenticity has largely succeeded in matching the challenges presented by consumer demand for original products. The tourism sector, for example, has benefited significantly. Unfortunately, there is little empirical evidence to support the bi-dimensionality of (subjective and objective) authenticity in the food sector. Thus, in contrast to what happened in the study of other topics or in other economic sectors, there exists no psychometrically validated measurement scales that is consistent with the theory of authenticity as a bi-dimensional construct. Therefore, the purpose of this study is to address this issue by developing and validating a measurement of consumers’ views of authenticity of traditional cheese specialties and to analyse both its antecedent, i.e., identification with the product, and its consequence, namely how it affects stated-willingness to consume.

## 2. Research Framework

**Figure 1:**
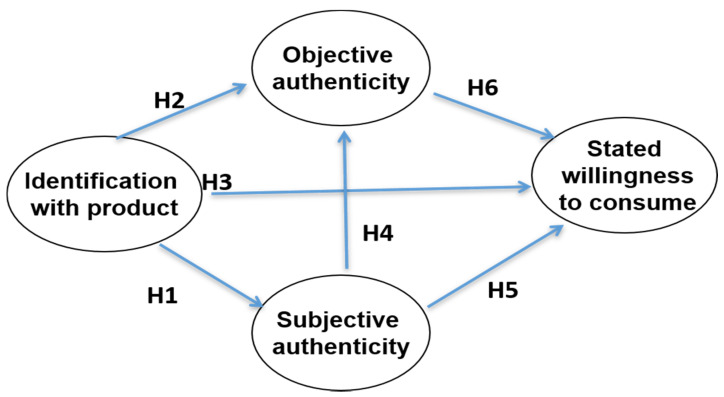
Research hypotheses.

The central role of identification with a product is often espoused in marketing literature. Lunardo and Guerinet [6] suggest that the relevance of a product depends on whether it is able to trigger its consumption by projecting the consumer’s personality. Furthermore, identity refers to upbringing, beliefs, stories, cultural ways of living and conceptions of what the product is to be [26]. Based on these factors, we postulate that (Figure 1):

**Hypothesis 1** **(H1).** The higher the identification with a product, the higher the subjective authenticity.

**Hypothesis 2** **(H2).** The higher the identification with a product, the higher the objective authenticity.

**Hypothesis 3** **(H3).** The higher the identification with a product, the higher the stated willingness to consume.

Based on Marsden [30], we also state that food is not simply a commodity, but a social construct, as well. As a consequence, this social construct reinforces the mental images of the perceived product’s setting, such as place, method of production, quality and time, among consumers. Thus, our fourth hypothesis is:

**Hypothesis 4** **(H4).** The higher the subjective authenticity elicited by a product, the higher the perceived objective authenticity.

According to Bemporad and Baranowski [31], consumers reward producers of authentic products by displaying a preference for these products. Moreover, Langen [27] proposes that social and environmental issues related to sustainably produced food can affect consumers’ decision-making. Therefore, we assume that authenticity has a positive impact on stated consumption behaviour. This leads us to hypotheses five and six:

**Hypothesis 5** **(H5).** The higher the subjective authenticity elicited by a product, the higher the stated willingness to consume.

**Hypothesis 6** **(H6).** The higher the objective authenticity elicited by a product, the higher the stated willingness to consume.

## 3. Methodology

### 3.1. The Research Steps

**Figure 2:**
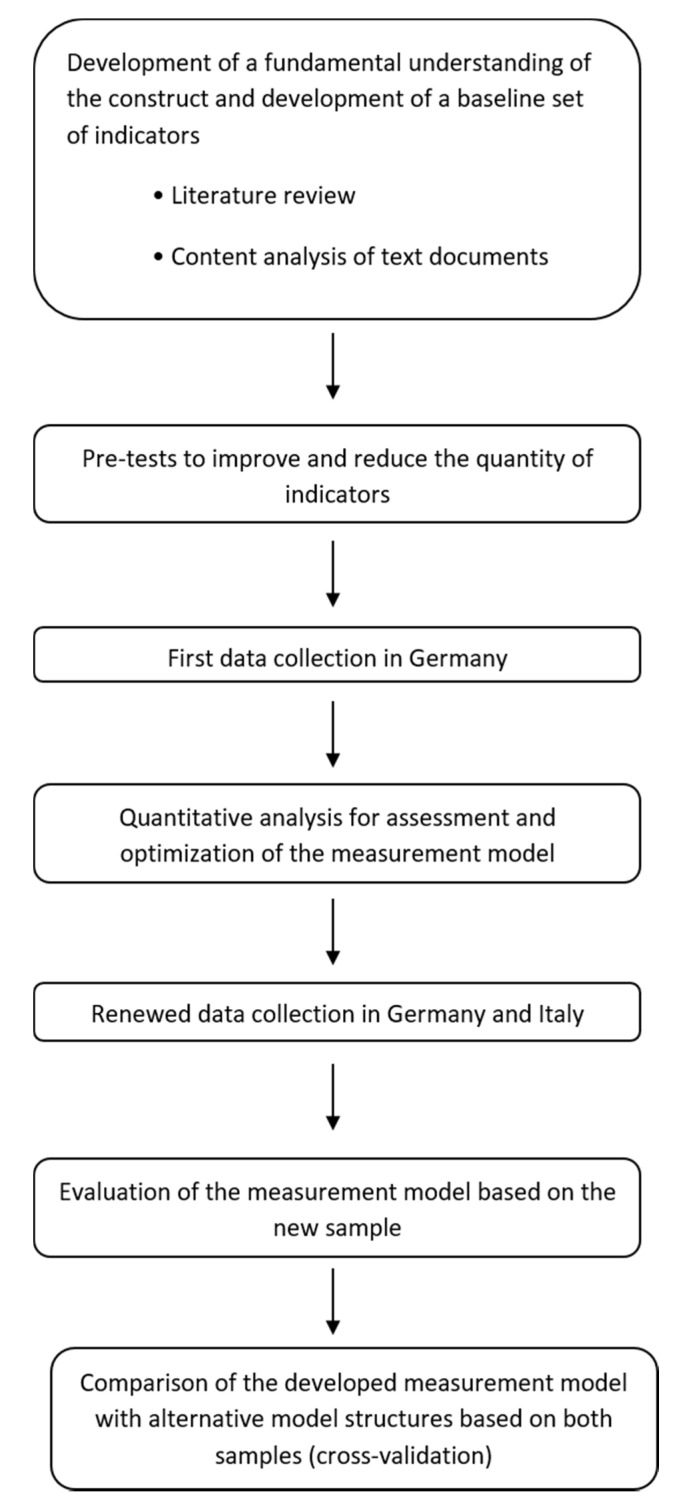
The research steps.

The scale construction and assessment techniques employed in this study follow those advocated by Gerbing and Anderson [32], Byrne [33], Homburg and Giering [34] and Brunsø et al. [35]. A seven-step procedure was implemented. The first step involved a review of the literature which led to the generation of a pool of items. The second step involved expert validation and the subsequent cleansing of some statements. A group of three experts in food marketing was asked to evaluate the accuracy of the items. They needed to reflect the factors of subjective and objective authenticity, identification with a traditional food specialty and stated willingness to consume. As a result of this step, the number of items included in the initial version of the measurement instrument was reduced. In the third step, the reduced measurement instrument was pre-tested. The survey was conducted in Germany in December 2012 with 136 German consumers. A private marketing research panel provider recruited the participants using three quotas in order to achieve a sample which was representative of the German population in the following aspects: age, gender and income. In the fourth step, we conducted an exploratory factor analysis (EFA) for assessing and optimizing the measurement model. This led to the further cleansing of some items and the introduction of new ones. In the fifth step, the cross-country study took place in Germany and in Italy. Since we strived for samples of consumers of traditional specialty foods, we applied the following filtering questions: (1) only respondents who consume cheese at least once a month were chosen and (2) we selected those respondents that declared, on a scale from 1 (never) to 5 (very often), to at least sometimes buy food from a specialty store, such as organic and farm shops, or delicatessens. Both the pre-test, as well as the 2013 questionnaire, were designed in German. The Italian version of the questionnaire was checked against the German version by translation and back translation by Italian and German native speaking researchers. As far as data collection is concerned, this step computed 220 complete responses, with a completion rate of 90.16% in Germany and 273 complete responses with a completion rate 90.75% in Italy. However, the total number of valid replies was 208 for the German survey and 200 for the Italian study, as survey responses finished in less than four minutes were cut off as untrustworthy. In the sixth step, we conducted an exploratory factor analysis for both the German and the Italian sample and compared the factor solutions across and within the samples. Finally, in the seventh step, we ran the final model, by means of confirmatory factor analysis (CFA), and we compared results across the German and the Italian sample by testing for cross-cultural validity of the scale measurement (Figure 2).

In all three surveys, respondents were asked to evaluate the two dimensions (subjective and objective) of authenticity concerning the PDO cheese Algovian Emmentaler (AE). AE is a well-known cheese made from the raw milk of cows that have been fed with grass and hay from the Algovian region in southern Germany. The decision to choose a traditional cheese as the object of our research is manifold: firstly, cheese belongs to the category Dairy Products, and dairy plays a fundamental role in the nutritional habits of Europeans [36]. Secondly, cheeses are the most represented traditional food speciality protected with the PDO label in Europe (http://ec.europa.eu/agriculture/quality/door/list.html?locale=de). Finally, this cheese specialty had almost disappeared during the 1990s due to the rapid increase in availability of the industrialized Emmentaler cheese, which is made with pasteurized milk [37,38]. Consequently, the production of Algovian Emmentaler is more expensive, due to the extra costs implicit in the artisanal mode of production. The PDO certification scheme helped Algovian Emmentaler producers to sell at a premium price and, in this way, to keep on producing it.

### 3.2. The Questionnaire

The questionnaire used in the surveys had four components: (a) perceived objective authenticity (methods of production, ingredients and origin of ingredients), (b) perceived subjective authenticity (idealization of agrarian life, solidarity with farmers and respect for natural biodiversity), (c) identification with the product (the potential of the product to elicit projective attributes in the consumer’s personality) and d) overall stated willingness to buy the cheese or to purchase it again (only one item). The questions were supplemented with a picture of the AE cheese. Being an artisanal product, the cheese was shown without packaging, as it is usually sold in delicatessen stores. However, the picture of the cheese was complemented by the red label of the PDO as is mandated by European regulation regarding geographical indications which obliges retailers to expose the labelling next to the products [39].

## 4. Results

### 4.1. Sample Description

Three online surveys were conducted, with the first taking place in December 2012 and comprising a panel of 136 German consumers. Three quotas were used to achieve a representative sample (Table 1): age, gender and income. Respondent ages ranged from 18 to 82, with an average of 49 years of age. In terms of gender, 48% of the respondents were male while 52% were female. Meanwhile, respondent incomes ranged from ca. €900 a month to over €4501. Using a 5-point scale ranging from 1 (never) to 5 (very often), mean cheese consumption was 3.06, showing a modest consumption of Algovian Emmentaler. However, after reading a description of Algovian Emmentaler, mean interest in purchasing it (“I’m going to buy this cheese in the future”) was higher, 3.96 with 1 meaning “strongly disagree”, 3 meaning “neither” and 5 meaning “fully agree to”. This survey served as a pre-test for the next cross-country survey that took place in Germany and Italy with samples of gourmet consumers. The two surveys were executed by a provider of survey panellists from 3 September 2013 to 9 September 2013. The total number of valid responses was 208 in the German sample and 200 in the Italian sample. The demographics of these German and Italian respondents are presented in Table 1.

The majority of German respondents (14.4%) were located in the German state of Baden-Württemberg, followed by 12% located in the German state of Bavaria and 7.7% in Berlin (data not shown). In contrast to the pre-test, in the German gourmet sample 47.8% were female and 52.4% were male. Similarly, in the Italian sample, 46.4% of the respondents were female while 53.6% were male. The majority of them (24.9%) were located in the region of Lombardy, followed by 11% who were located in the region of Campania and 7.2% in the region of Lazio. Average ages were also similar between the two countries; 53 years old in the German sample and 50.6 years old in the Italian one, with the youngest respondent being 30 and the oldest being 81 years old. In terms of monthly household income, in both samples dominated the income ranges between ca. €2001 and €3200.

### 4.2. Scale Construction: Exploratory and Confirmatory Factor Analyses

#### 4.2.1. Exploratory Factor Analyses

Separate exploratory factor analyses (EFA) with principal component analyses and Varimax rotations were carried out for each sample. The aim of this procedure was to check whether items would tend to group together in similar factors across the three samples, which then led to the rejection of some items. Appendix A presents the items obtained through the EFA and how they loaded on the respective factor in each of the three samples, showing in bold which items were used in the confirmatory factor analysis (CFA) that followed. Regarding objective authenticity, items 1–4 showed constantly loading values higher than five both across time (in the pre-test of 2012 and German survey of 2013) and across countries (Germany and Italy). The item “Algovian Emmentaler comes from the best-known country in the world for the production of this type of cheese” was introduced after the pre-test, so its loading was only calculated for the cross-country surveys, while another item was loaded in a different factor. Interestingly, items 5–11 displayed consistent patterns only in the pre-test but not in the second pair of surveys with gourmet consumers. Therefore, they were not kept for further analysis. Concerning subjective authenticity, items 1–5 also showed consistent loading values higher than five across time, while items 6–9 displayed no clear pattern and were thus not further considered for the CFA. In terms of identification, items 1–4 were used for the following CFA, whereas items 5–8 were excluded. However, two items in the identification factor were only introduced after the pre-test: “The consumption of this product contributes to the improvement of my wellbeing” and “This cheese is the pride of a culture with which I can identify myself.” All in all, three factors were identified that appeared to be constant across the three samples: objective authenticity, subjective authenticity and identification. As shown in the next section, those items with loadings consistently high in all three samples were combined into scales.

#### 4.2.2. Confirmatory Factor Analyses

Originally, there were 33 indicators spread over five factors. Following the EFA, this number was narrowed down to 13 indicators in three factors (Table 2).

The objective authenticity factor contained four items, two of which referred to the traditional method of production and the region of production. The latter is particularly important, as it indicates not only the origin of the specialty’s ingredients but its reputation, as well, which is a fundamental requisite when obtaining protected labels of designation of origin.

Subjective authenticity contained five items highlighting the social and economic sustainability values attached to the product, such as feeling close to the producers, allowing the producer to express themselves, and supporting the region economically. Another item revealed consumers’ idealization of niche products originating from rural areas, through their “moral purity” versus mass-produced products.

Finally, the identification factor consisted of four items disclosing how the consumer identifies with the product personally, symbolically, culturally and in terms of their well-being. Factors related to objective and subjective authenticity were found to share similar tendencies across all three samples. Both gourmet samples identified more with Algovian Emmentaler than the pre-test sample. Expectedly, the identification factor’s values also scored higher among the German respondents, as the Italian respondents identified less with Algovian Emmentaler.

Confirmatory factor analysis was conducted using the software AMOS 21. CFA indicates a special case of the general model of causal analysis, which is identified in more precise formulation as covariance structure analysis [34]. A complete causal analysis model consists of two components:

• The measurement model:

Through confirmatory factor analysis, it shows how the indicators explain the latent variables (factors).

• The structural model:

With the help of structural equation analysis, it shows the explanation of the exogenous variables by the endogenous latent variables (factors).

Furthermore, Ref. [41] demonstrated that CFA can be used to determine whether a set of data is compatible with a pre-specified factor structure. It can also be applied to multiple samples, and then used to confirm whether the factor structure in each sample is the same. This allows the reliability of a factor to be examined, as well as how well it is measured together with its associated indicators [34]. In the present case, all the factors had good factor reliability (FR), i.e., composite reliability, and average variance extracted (AVE) values (FR: > 0.6 and AVE: > 0.5), as is found in Table 3.

In covariance-based structural equation modelling the adequacy of the model is measured by a range of goodness-of-fit criteria (see Table 4). Using different indices is considered by many scholars the best strategy to overcome the limitations of each index [33,34].

The overall fit met the conventional cutoff-criteria in all three samples [34]. Furthermore, the best scores in terms of Root Mean Square Error of Approximation (RMSEA) and Goodness-of-Fit Index (GFI) were those displayed in the pre-test and the Italian samples. On the contrary, the German sample scored best with regards to the Comparative Fit Index (CFI) and the Tucker–Lewis Index (TLI).

#### 4.2.3. Structural Equation Model

Using the validated authenticity scale produced by the confirmatory analysis and the identification factor, evidence was found of the influence of consumers’ perceptions of authenticity and identification with a specialty product on their purchasing intentions. Furthermore, using the software AMOS 21, identical models were built based on the two samples obtained by the cross-country surveys (Germany and Italy). The robustness of the theoretical framework was tested with covariance-based structural equation modelling (SEM). According to Byrne [33], the RMSEA is one of the most informative criteria in assessing the fit of the structural equation model. As already shown in Table 4, both the Italian and the German sample had adequate RMSEA scores (0.046 vs. 0.081, respectively) when judged based on the recommended cut-off criteria (values less than 0.05 indicate good fit, and values as high as 0.08 represent reasonable errors of approximation in the population. Whilst RMSEA values ranging from 0.08 to 0.10 are still acceptable, those greater than 0.10 indicate poor fit).

Both German and Italian models comprised one exogenous latent variable (identification with the product) and three endogenous latent variables (subjective and objective authenticity, as well as stated willingness to consume).

#### 4.2.4. Outlook

**Figure 3:**
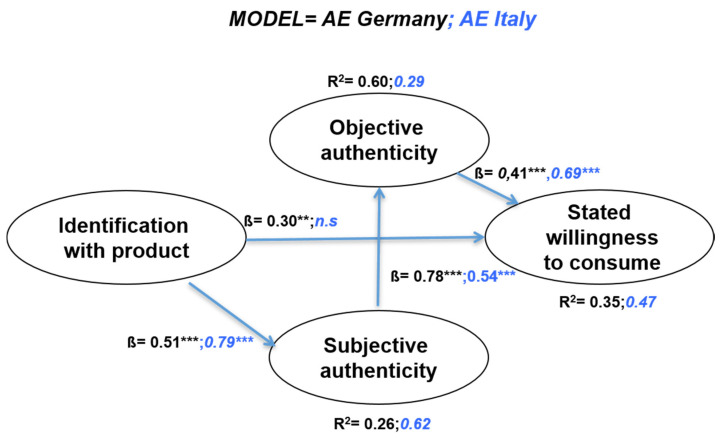
Results of the two structural equation models; *** = *p* < 0.001, ** = *p* < 0.01, n.s. = non statistically significant.

Identification with Algovian Emmentaler directly accounted for 26% of the explained variance of the subjective authenticity in Germany and 62% in Italy (Figure 3). Subjective authenticity was directly responsible for the majority of objective authenticity’s variation in both samples, albeit more so in the German sample’s case (60%) than in the Italian sample (29%). Finally, objective authenticity-together with identification-explained 47% of the variation of the stated willingness to consume in the Italian sample, followed by 35% in the German one.

The hypotheses were tested by examining the sign, size and statistical significance of the structural coefficients. The hypotheses regarding the relationship among the constructs tested in the final model were partially accepted in both models. In the following, only direct effects are taken into consideration. The parameter estimates for the relationship between identification and subjective authenticity were statistically significant and consistent with the proposed direction of our first hypothesis (H1) in both the German and Italian samples (ß = 0.51 *** and ß = 0.79 ***, respectively). However, this does not hold true for the effect of identification on objective authenticity—H2 was rejected in both samples.

In terms of the relationship between subjective authenticity and objective authenticity, the path coefficient was statistically significant and consistent with the proposed direction of our fourth hypothesis. H4 was accepted in both samples, with ß = 0.78 *** in the German sample and ß = 0.54 in the Italian sample. Contrarily, the effect of subjective authenticity on the stated willingness to consume (H5) was not statistically significant in either of the two samples, and thus not accepted. Objective authenticity’s effect on stated willingness to consume was relatively strong and statistically significant in both Germany and Italy (ß = 0.41 *** and ß = 0.69 ***, respectively; H6 was accepted). Finally, the expected effect of identification on the stated willingness to consume was significant and positive in Germany (ß = 0.30 **), but not in Italy; therefore, H3 was accepted in the German sample and not accepted in the Italian sample. The latter’s rejection is expected considering Algovian Emmentaler’s origin is outside of Italy.

Overall, the subjective dimensions of authenticity were found to be consistently higher in Germany than in Italy. This, along with the high direct effect of identification, could be interpreted as a higher emotional anchorage of consumers in Germany with “their” food specialty compared with the Italian sample. Conversely, the effects of the objective dimensions of authenticity on stated willingness to consume were higher in Italy, which may be caused by the country’s more developed culinary culture and the generally higher knowledge of GIs. In a nutshell, both models explained significant amounts of the variation in the endogenous variables.

## 5. Discussion

The objective of this research was to build an authenticity scale of traditional cheese specialties and we tested it in two regions that are positioning themselves as food destinations in Germany and in Italy. Another goal of this work was to explore the effect of customers’ perception of authenticity—whether it is perceived subjective authenticity or objective authenticity—and their identification with a product on their stated willingness to consume it, as well as how this effect varies across countries. Using a sample of consumers of specialty foods from the above-mentioned countries, the findings of our model generally showed that objective and subjective authenticity as well as identification with a product affected stated willingness to consume. Objective authenticity had a direct influence on stated willingness to consume in both countries. This was also a mediator of subjective authenticity, while the latter was a mediator of the effect of identification on objective authenticity. Unsurprisingly, only in the German sample did respondents’ identification with Algovian Emmentaler have a direct effect on their stated willingness to consume it.

This finding confirms previous literature. For instance, several studies showed that consumers prefer products that reflect their actual or desired personality [44,45,46]. Niinimaki [47] affirmed that consumers continuously construct their identity through the consumption of products that reflect their personality. In this sense, a person’s identification with the profile of green consumers positively influences his/her intention to purchase environmentally friendly products [48,49]. As such, being a German specialty, the sample of German consumers could identify with this cheese, but not the Italian one, although both samples considered the cheese specialty to be authentic. Furthermore, being held across two different timeframes and with a cross-country design, our scale seems to suggest that a bi-dimensional construct might be a suitable means to measure authenticity”.

While the proposed model shows that subjective authenticity is clearly linked to consumers’ ethical and sustainability-related attributes (Table 2), objective authenticity displays a direct and measurable effect on the stated intention to consume the food specialty. As mentioned before, the construct of objective-authenticity consists of items that range from respect for the tradition, to the unique connection of the product with the place of origin (Table 2). Thus, this dimension reflects the rationale of country-of-origin labels such as PDO, Slow Food, etc., which identify a food with its origin and link food quality with its geographical environment, including natural and human factors [39]. In line with the results of Sidali and Hemmerling [25], this finding shows once again the reassuring importance of food claims on credence attributes of food.

Modern consumers purchase foods from certified sources in order to access high-quality foods and to contribute to environmental and society-friendly agriculture. However, as remarked by De Haen and Réquillart [50], an important precondition for consumers to express their preferences for food from sustainable production is availability of objective information about the product and about process quality. Hence, policy intervention is called for to find a balance between the right of consumers to be adequately informed, and in this way reduce their food illiteracy, and the right of the different stakeholders along the food supply chain to run their businesses with the right amount of ‘entrepreneurial freedom’ [50]. In other words, on the one hand, farmers and/or processors are already overtasked by burdensome rules and standards, to the extent that it appears unrealistic to expect them to accept further rules or certification schemes [50], but on the other hand, many scholars agree on the necessity of investing in equity and sustainability in modern agricultural systems as a moral societal task [51].

It is the opinion of the authors that country-of-origin such as PDO and PGI seem to be effective means of conveying and reinforcing ethical and sustainability-related values, as well as promoting best practices among agricultural food systems. GIs stem from the necessity of reducing moral hazards in the way food is produced and consumed when traded in the international marketplace, thus protecting both producers and consumers from counterfeiting and fraud. As mentioned earlier in our study, governmental support towards such specific certification schemes was successful in the past in helping those producers who could not, or did not want to, opt for a product reformulation (pasteurized milk instead of raw milk; forage instead of hay)—a recognized business practice of the food industry, [51]—and enabled the emergence of long-term societal benefits with regard to both product quality and consumer empowerment.

Furthermore, governmental support towards such schemes helps counterbalance the downstream shift in the drivers of product differentiation, to the benefit of upstream producers, as is shown in the case of wine production by [50]. A targeted and exemplary policy to increase GI schemes has been offered through a piece of legislation in Italy. In order to boost regional gastronomy, farms offering agrotourism are called upon to offer mostly locally produced food products to their guests. Furthermore, to promote Italian regional gastronomic traditions, the law recommends the provision of foods with protected quality names such as PDO and PGI. In order to obtain ‘new relationships between agriculture and society’ [52], policy-makers need to facilitate the promotion of alternative food supply chains, even if this means a rethinking of those food policies of Western governments that have aimed to keep food prices low at all costs. The cost of tracking food specialties is higher compared to the cost of producing a “placeless” product [53]. However, the statistically significant effect of perceived authenticity on stated willingness to consume displayed in our model indicates that consumers of traditional food specialties are ready to pay a higher cost, if they perceive these products as authentic. This is also reflected in the literature: on average consumers honour producers’ higher costs by paying a higher premium that can range from an average of 21% [54] to up to 300% [53], for a specialty with a country-of-origin label. At an aggregated level, this means an empowerment of consumers who are less distant from the locus of food production, as well as a redistribution of profits along the food chain from those who sell on a large scale to those who grow the food [51].

## 6. Conclusions

Prior studies have documented the importance of analysing the potential perception of food products and have called for more work to analyse the communication of food distinctiveness in both qualitative and quantitative ways [55]. In this article, the authors have built an authenticity scale of food specialties using a cross-country design and have tested the scale in two different timeframes in order to facilitate the comparison of results. The findings of this study will be helpful for producers of certified foods by helping them to improve their understanding of consumers’ specific views on authenticity, along with which dimensions of authenticity—subjective or objective authenticity—most strongly affect their stated willingness to consume specialty products. These findings also provide a basis for marketing strategies that emphasize the origins of products and can help policy-makers to develop national food policies. As an avenue for future research, it would be interesting to analyse whether the proposed scale of perceived authenticity also holds true for other categories of traditional food specialties such as fresh fruits and vegetables or meat products. This study does contain limitations as well. Although traditional food specialties are at the heart of European Union initiatives in the food sector, rural areas host even richer homemade, local and varied produce, which has been neglected by the authors in this current study. To survey consumer understanding of such embedded, often artisanal food, the current work could have integrated the authenticity scale with qualitative research designed to unearth different levels of awareness of such products among consumers. This would have provided the reader with a more complete picture of consumers’ cognitive and affective dimensions of territorialized food. All in all, the proposed scale is a departure point for further research. The authors are aware that the validity of the current work remains in the hands of the research community who may replicate this instrument and further define the extent of appropriateness of this measure for future studies on authenticity in the food sector.

## Figures and Tables

**Table 1:** Socio-demographics of the pre-test (n = 136), German (n = 208), and Italian (n = 200) samples.

*Variable*	*Survey Response*	*Pre-Test Survey (% unless Otherwise Stated)*	*German Survey (%)*	*Italian Survey (%)*
Gender	Female	52	47.6	46.4
	Male	48	52.4	53.6
Age	<50	47.5	46.2	48.8
	51–70	42.6	45.2	47.4
	71 or older	9.6	8.6	3.8
	Average age, years	49	53	50.6
Household composition	1 member	30.1	21.2	10.5
	2 members	41.9	44.7	28.7
	3 members	15.4	16.8	29.7
	4 or more members	12.5	17.3	31.1
Monthly income per household	Less than €900	13.9	3.4	7.2
	€900–€1500	25.5	15.4	18.2
	€1501–€2000	16.1	15.9	22.5
	€2001–€3200	24.5	33.2	26.8
	€3201–€4500	12.0	23.1	17.2
	€4501 or more	8.0	9.1	8.1

**Table 2:** Confirmatory factor analysis.

Factor	Item	Factor Loadings/Item Reliability		
		Pre-Test	Germany	Italy
Objective Authenticity	I can trust the name AE.	0.909/0.827	0.830/0.689	0.829/0.688
	AE cheese-makers are very oriented toward respect for tradition.	0.754/0.569	0.783/0.614	0.811/0.658
	AE comes from the best-known country in the world for the production of this type of cheese.	*	0.706/0.499	0.770/0.593
	I am sure that all the ingredients of this product come from the Algovian region.	0.716/0.512	-	0.723/0.523
Subjective Authenticity	AE makes me feel close to the producer.	0.756/0.571	0.789/0.622	0.841/0.708
	AE allows producers to charge fair prices.	0.742/0.550	0.763/0.582	0.758/0.575
	AE is the embodiment of moral purity.	0.790/0.624	0.813/0.660	0.749/0.560
	AE allows the cheese-makers to express themselves and be creative.	0.760/0.577	0.731/0.534	0.766/0.587
	AE supports the region economically.	0.773/0.598	0.722/0.521	0.654/0.428
Identification	This cheese fits my lifestyle and the way I shop.	0.902/0.814	0.884/0.782	0.808/0.653
	The consumption of this product contributes to the improvement of my wellbeing.	*	0.793/0.629	0.873/0.763
	This cheese is the pride of a culture with which I can identify myself.	*	0.842/0.709	0.779/0.607
	I can identify with this cheese.	0.758/0.575	0.879/0.773	0.816/0.666
Intention to consume	I will definitely (continue to) eat Algovian Emmentaler in the future.	-/0.59	-/0.35	-/0.47

AE = Algovian Emmentaler cheese * = Item was not given in the pre-test; - = Item did not load on the same factor. Sources of items: Objective authenticity, 1. based on Liao and Ma [22], 2. based on Beverland [5], 3. based on Camus [40], 4. own item; subjective authenticity, 1. and 2. own item, 3. based on Liao and Ma [22], 4. and 5. own item; identification, 1. based on Grunert et al. [41], 2. based on Davis [42], 3. based on Carstensen [43], 4. based on Camus [40]; intention to consume: own item (for one-item statements only item reliabilities are calculated).

**Table 3:** Composite reliability.

Factor	Factor Reliability/AVE		
	Pre-Test	Germany	Italy
**Objective Authenticity**	0.84/0.64	0.83/0.56	0.86/0.62
**Subjective Authenticity**	0.88/0.58	0.88/0.58	0.87/0.57
**Identification**	0.82/0.69	0.91/0.72	0.89/0.67

**Table 4:** Model fit (measurement and structural equation models).

Survey	Model Fit					
	Cmin/DF	CFI	RMSEA	GFI	NFI	TLI
Pre-Test	1.036	0.998	0.016	0.946	0.953	0.998
Germany	2.353	0.941	0.081	0.883	0.902	0.927
Italy	1.420	0.980	0.046	0.929	0.936	0.976
Cut-off criteria	1–3	0.90–0.95	≤0.05 very good ≤0.08 good	0.90–0.95	0.90–0.95	0.90–0.95

Legend: Cmin/DF = Chi-Squared/Degrees of Freedom; CFI = Comparative Fit Index; RMSEA = Root Mean Square Error of Approximation; GFI = Goodness-of-Fit Index; NFI = Normed Fit Index; TLI = Tucker–Lewis Index.

## Abbreviations

The following abbreviations are used in this manuscript:

SEM	Structural Equation Modelling
PDO	Protected Denomination of Origin
PGI	Protected Geographical Indication
GIs	Geographical Indications
Cmin/DF	Chi-Squared/Degrees of Freedom
CFI	Comparative Fit Index
RMSEA	Root Mean Square Error of Approximation
GFI	Goodness-of-Fit Index
NFI	Normed Fit Index
TLI	Tucker–Lewis Index
PDO	Protected Designation of Origin

## Appendix A

Exploratory factor analysis. Items in bold were used in the confirmatory factor analysis described in the article, while the others were excluded.

**Factor**	**Item**	**Pre-Test**	**Germany**	**Italy**
Objective Authenticity	**I can trust the name AE.**	-	0.733	0.775
	**AE cheese-makers are very oriented toward respect for tradition.**	0.581	0.701	0.799
	**AE comes from the best-known country in the world for the production of this type of cheese.**	*	0.696	0.765
	**I am sure that all the ingredients of this product come from the Algovian region.**	0.775	0.656	0.738
	AE cheese-makers are not mass-production companies.	-	0.625	0.574
	I can imagine where this cheese was produced.	0.742	-	-
	I can imagine how this cheese is produced.	0.712	-	#
	I am certain that this cheese is produced using traditional methods.	0.720	#	#
	I am certain that the quality of this product is above average.	0.702	#	-
	This cheese does not seem to contain artificial additives.	0.588	#	-
	I am certain of the origin of this product.	0.578	#	-
Subjective Authenticity	**AE makes me feel close to the producer.**	0.616	0.805	0.836
	**AE allows producers to charge fair prices.**	0.622	0.713	0.784
	**AE is the embodiment of moral purity.**	0.646	0.677	0.745
	**AE allows the cheese-makers to express themselves and be creative.**	0.512	0.588	0.743
	**AE supports the region economically.**	0.775	0.767	0.576
	AE is trustworthy	0.720	0.774	#
	AE tastes like all of the “earlier” products and is certainly better than conventional products	#	0.734	#
	AE is traditionally hand-made.	0.722	0.714	#
	This cheese protects the biodiversity of the Algovian region	0.660	#	-
Identification	**This cheese fits my lifestyle and the way I shop.**	0.788	0.840	#
	**The consumption of this product contributes to the improvement of my wellbeing.**	*	0.839	#
	**This cheese is the pride of a culture with which I can identify myself.**	*	0.782	#
	**I can identify with this cheese.**	0.750	0.811	#
	AE makes me smile because it is a product from my childhood.	#	0.709	#
	It doesn’t bother me that the taste of this cheese can vary from day to day like any other product made by hand.	0.605	0.590	-
	I find this cheese particularly attractive because you cannot find it in any store.	0.750	#	0.824
	This cheese expresses who I am.	-	#	-
Legend: Allgäuer Emmentaler = Algovian Emmentaler. * Item was not given in the pre-test. - Item did not load on same factor. # Item loaded to more than one factor or displayed loading < 0.5. Stated intention to consume Algovian Cheese was measured by the following statement: I will definitely (continue to) eat Allgäuer Emmentaler in the future.				
